# Auditory cortex modelled as a dynamical network of oscillators: understanding event-related fields and their adaptation

**DOI:** 10.1007/s00422-022-00936-7

**Published:** 2022-06-20

**Authors:** Aida Hajizadeh, Artur Matysiak, Matthias Wolfrum, Patrick J. C. May, Reinhard König

**Affiliations:** 1 Research Group Comparative Neuroscience, Leibniz Institute for Neurobiology, Brenneckestraße 6, 39118 Magdeburg, Germany; 2 Weierstrass Institute for Applied Analysis and Stochastics, Mohrenstraße 39, 10117 Berlin, Germany; 3 Department of Psychology, Lancaster University, Lancaster, LA1 4YF UK

**Keywords:** Auditory cortex, Event-related field, Adaptation, Synaptic depression, Normal modes, Slow-fast dynamics

## Abstract

Adaptation, the reduction of neuronal responses by repetitive stimulation, is a ubiquitous feature of auditory cortex (AC). It is not clear what causes adaptation, but short-term synaptic depression (STSD) is a potential candidate for the underlying mechanism. In such a case, adaptation can be directly linked with the way AC produces context-sensitive responses such as mismatch negativity and stimulus-specific adaptation observed on the single-unit level. We examined this hypothesis via a computational model based on AC anatomy, which includes serially connected core, belt, and parabelt areas. The model replicates the event-related field (ERF) of the magnetoencephalogram as well as ERF adaptation. The model dynamics are described by excitatory and inhibitory state variables of cell populations, with the excitatory connections modulated by STSD. We analysed the system dynamics by linearising the firing rates and solving the STSD equation using time-scale separation. This allows for characterisation of AC dynamics as a superposition of damped harmonic oscillators, so-called normal modes. We show that repetition suppression of the N1m is due to a mixture of causes, with stimulus repetition modifying both the amplitudes and the frequencies of the normal modes. In this view, adaptation results from a complete reorganisation of AC dynamics rather than a reduction of activity in discrete sources. Further, both the network structure and the balance between excitation and inhibition contribute significantly to the rate with which AC recovers from adaptation. This lifetime of adaptation is longer in the belt and parabelt than in the core area, despite the time constants of STSD being spatially homogeneous. Finally, we critically evaluate the use of a single exponential function to describe recovery from adaptation.

## Introduction

Most sounds, speech sounds in particular, make sense only when perceived against the backdrop of what came immediately before, in a time window extending some seconds into the past—the so-called psychological present (Michon 1978). The task of the auditory system is to retain information and to integrate it with representations of incoming stimuli. This process of memory and integration is likely to occur in auditory cortex, whereas the subcortical auditory pathway carries out the analysis of spectral structure and sound source localisation (Nelken 2004). While our understanding of the functioning of auditory cortex is limited, especially in comparison to that of the visual cortex (King and Nelken 2009), a number of memory phenomena have been identified in auditory cortex that operate on the time scale of hundreds of milliseconds to seconds. As reviewed below, these include context-sensitivity: the dependence of a neuronal response not just on the eliciting stimulus but also on preceding stimuli—the historical context. Further, the memory phenomena observed in the physiological responses of auditory cortex have been linked to behaviourally measured sensory memory (Tiitinen et al. 1994) and working memory (Brechmann et al. 2007; Huang et al. 2016).

The simplest form of context sensitivity can be observed by repeating the stimulus within a time window on the order of seconds. The repeated stimulus elicits an auditory response with a reduced amplitude, with the reduction tending to be inversely related to the stimulus onset interval (SOI). This phenomenon is called adaptation, and it is also known as repetition suppression or habituation (Megela and Teyler 1979; Pérez-González and Malmierca 2014). Adaptation is observed in both non-invasive and invasive measurements. When adaptation does not generalise to all stimuli, a neuron can produce a large-magnitude response to a stimulus that is different from the repeated one. This is known as stimulus-specific adaptation (SSA), a phenomenon seen in the primary auditory cortex of animal models (Ulanovsky et al. 2003, 2004). In magnetoencephalography (MEG) and electroencephalography (EEG) measurements in humans, adaptation manifests itself most clearly in variations of the most prominent auditory evoked response, the N1m or N1, respectively. Here, we use N1(m) to denote both the MEG and EEG variety of the response. Several studies have shown that the peak amplitude of the N1(m) attenuates with stimulus repetition and is inversely related to stimulation rate (see, for example, Davis et al. 1966; Hari et al. 1982; Imada et al. 1997; Ioannides et al. 2003; Loveless et al. 1996; Lü et al. 1992; Lu et al. 1992; McEvoy et al. 1997; Megela and Teyler 1979; Nelson et al. 1969; Sable et al. 2004; Wang et al. 2008; Zacharias et al. 2012). Further, this adaptation develops rapidly in that already the second stimulus elicits a diminished response (e.g., Budd et al. 1998; May and Tiitinen 2004; Rosburg et al. 2010). The monotonic increase of the N1(m) amplitude as a function of SOI which then plateaus out at large SOI (>5 s) can be approximated with an exponentially saturating function. The time constant of this function provides an estimate of the time span it takes for the auditory system to recover from adaptation (Lü et al. 1992; Lu et al. 1992). The picture becomes complicated when short SOIs of under 500 ms are used. In this case, the SOI dependence of the N1(m) amplitude can take on a non-monotonic v-shape (Budd and Michie 1994; Wang et al. 2008; Zacharias et al. 2012), and the rapid presentation of tones elicits a sustained response upon which the diminished N1(m) responses ride (May and Tiitinen 2004, 2010).

Adaptation due to stimulus repetition can be found in all parts of the auditory system. It tends to have a shorter recovery time subcortically than in cortex, especially in the lemniscal pathway (for a review, see, Pérez-González and Malmierca 2014). For example, the auditory nerve recovers within 25-35 ms (Yates et al. 1983). In the superior olivary complex, both the onset and the recovery from adaptation have respective time constants of 20 ms and 106 ms (Finlayson and Adam 1997). Studies using relatively high stimulus rates of above 3 Hz have reported adaptation in the inferior colliculus (IC) (Palombi and Caspary 1996; Nuding et al. 1999). For the majority of units in the IC, SSA requires SOIs shorter than 250 ms (Pérez-González et al. 2005; Malmierca et al. 2009). However, Zhao et al. (2011) observed SSA in the IC even with a SOI of 1 s, although these authors were not able to determine whether the units were in the lemniscal or non-lemniscal pathway. The lemniscal division of the auditory thalamus shows SSA only with SOIs shorter than 250 ms, although SSA could be observed in the non-lemniscal thalamus even with SOIs of up to 2 s (Antunes et al. 2010). Neurons in auditory cortex display SSA with SOIs up to several seconds (Tasseh et al. 2011).

The most plausible mechanism underlying cortical adaptation is short-term synaptic depression, STSD (Wehr and Zador 2003, 2005), a form of synaptic plasticity based on vesicle depletion as well as inactivation of release sites and calcium channels (Fioravante and Regehr 2011). This type of plasticity has decay times of hundreds of milliseconds to several seconds, and this coincides with the time course of cortical adaptation (Ulanovsky et al. 2004). STSD is also present in subcortical stations (for a review, see, Friauf et al. 2015). STSD can contribute to efficient information transfer between two neurons (Benda et al. 2005; Salmasi et al. 2019), to temporal filtering occurring at synapses (Fortune and Rose 2001; Rosenbaum et al. 2012), and to gain control (Abbott et al. 1997). Also, recovery from STSD during communication pauses has been linked to maximising the effect of post-pause communication signals (Kohashi et al. 2021). Computational models show that STSD accounts for different forms of context sensitivity in the AC (Loebel et al. 2007; May and Tiitinen 2010; Mill et al. 2011; Wang and Knösche 2013; May and Tiitinen 2013; Yarden and Nelken 2017; Kudela et al. 2018). Further, simulations show that STSD can function as a memory mechanism that allows for the representation of temporally extended, complex auditory information such as speech and species-specific communication sounds (David and Shamma 2013; May and Tiitinen 2013; Motanis et al. 2018). In sum, rather than signifying mere neuronal fatigue, adaptation might instead reflect the fundamental dynamics of synaptic depression which endows the auditory cortex with the ability to represent auditory information across different time scales (Benda 2021).

Adaptation of the N1(m) has been linked to information processing in auditory cortex. The recovery time from adaptation is a subject-specific parameter, and it correlates well with the time span of working memory in a forced-choice discrimination task (Lü et al. 1992; Lu et al. 1992). Adaptation is also the major determinant in the evoked responses elicited in the oddball paradigm. Here, standard stimuli presented with a high rate elicit a smaller event-related field (ERF) response than the infrequent deviant stimuli (Butler 1968), and the difference in response amplitude is termed the mismatch negativity (MMN) (Näätänen et al. 1978). The mismatch response is brimming with functional significance: it might serve the orienting reflex, it is linked to a large number of memory and learning phenomena, and it is altered in several clinical conditions ( for reviews, see, Näätänen 1990, 1992; May and Tiitinen 2010). Adaptation is likely to be at the root of the mismatch response both directly and indirectly. First, the differential between the deviant and standard response reflects the high and low level of adaptation of the N1(m) response elicited by the standards and deviants, respectively, due to their different presentation rates (Butler 1968; May et al. 1999; May and Tiitinen 2010). Further, May et al. (2015) used simulations of auditory cortex to show that short-term synaptic depression has multiple consequences: not only does it cause the adaptation of the response elicited by the frequently presented standards, but it could be the mechanism which integrates auditory information across time more generally. This integration shows up as context-sensitive single- and multi-unit responses to tone pairs (Brosch et al. 1999; Brosch and Schreiner 2000), and as mismatch responses in the ERF to deviations in tone-sequence structure (Näätänen et al. 1993). In this view, STSD not only underlies repetition suppression and the mismatch response, but it also allows the auditory cortex to represent complex, temporally-evolving sounds. Note that the adaptation-related explanation of the mismatch response (May and Tiitinen 2010; May et al. 2015) is only one alternative. Näätänen (1990, 1992) proposed that MMN is unrelated to adaptation of the evoked response, instead reflecting a process separate from that generating the N1(m). Also, the currently dominant predictive coding explanation suggests that repetition suppression is due to a top-down, inhibitory prediction signal matching the bottom-up sensory signal, and that the MMN is an indication of prediction error when the two signals do not match (Friston 2005; Bastos et al. 2012) (however, see, Rescorla 2021; May 2021).

Observing adaptation in the human brain generally requires the use of non-invasive techniques. MEG and EEG are well suited to this because they have a high temporal resolution of milliseconds, the time scale of neuronal responses. However, these methods have the drawback that it is difficult to identify the sources of the activity and their distribution. The response to a stimulus represents the simultaneous activation of around a million synapses on pyramidal cells forming an intricate network across auditory cortex, but what we observe is a spatial average of this activity (Hämäläinen et al. 1993). Therefore, MEG and EEG measurements in themselves reveal very little of the underlying neuronal dynamics. One way to move forward beyond observation is to use computational modelling. The aim of such modelling needs not be a faithful reproduction of the brain. Instead, modelling attempts to explain experimental observations by capturing the key mechanisms of the system under investigation. While no model should be required to duplicate the modelled system, a useful model is a device which reveals something about the system which would otherwise remain hidden, buried in the experimental data.

In our previous work (Hajizadeh et al. 2019, 2021), we sought to understand the generation of the event-related field in terms of a dynamical system with the spatial organisation of the auditory cortex (Kaas and Hackett 2000; Hackett et al. 2014). Our starting point was the model introduced by May and Tiitinen (2013) and May et al. (2015). This describes auditory cortex as a system of hundreds of units representing cortical columns, distributed across multiple fields in the core, belt, and parabelt areas. Synaptic strengths are dynamically modulated by STSD so that the interactions between the units become dependent not only on the current stimulus but also on the stimulation history. As explained above, this model captures the phenomenology of context sensitivity of auditory responses. However, it is highly non-linear and analytically impenetrable, and can only be studied one simulated trajectory at a time. Therefore, in Hajizadeh et al. (2019), we made the original model tractable by assuming that the input-output relationship of the model unit is linear and by using fixed connection strengths thereby ignoring the effects of STSD. This allowed us to study the explicit solutions of the dynamical equations of the model and to characterise the system in terms of its oscillatory properties. In this view, while the cortical column is the system’s structural unit, the dynamical building block is the normal mode: a damped harmonic oscillator emerging out of coupled excitation and inhibition.

The approach in Hajizadeh et al. (2019), which we also adopt here, is not just to replicate the ERF so as to explain it in terms of parameter dependencies. Rather, we are exploring and interpreting ERF generation in the context of a fundamentally new view on AC activity: First, auditory cortex behaves as a set of independent oscillators—normal modes—each characterised by a specific frequency, decay rate, and spatial profile. These oscillators do not reside in any one unit of auditory cortex but, rather, each oscillator is spread across the whole auditory cortex. Conversely, the activity of each unit is a weighted superposition of all the normal modes of the system. Second, the neural activity observed at the local level of individual columns as well as that observed on the global level as the ERF are both explicitly dependent on the anatomical structure of the entire AC. Third, the spatiotemporal pattern of the cortically generated ERF represents a superposition of all the oscillating normal modes. The ERF in the normal-mode picture is therefore fundamentally a system property of the *whole AC*. In a sense, this interpretation is an alternative to the classical equivalent-current dipole (ECD) description of discrete (see, for example, Scherg 1990; Mosher et al. 1992; Scherg and Berg 1996) and distributed (see, for example, Dale and Sereno 1993; Hämäläinen and Ilmoniemi 1994; Mosher and Leahy 1999) source modelling approaches in which ERFs are considered to arise from a linear sum of *multiple spatially distributed point-like sources (ECDs)*. The magnetic fields generated by ECDs are determined by the physics of electromagnetic fields described by the quasi-static Maxwell’s equations. Importantly, the ECD approach does not account for the dynamical interaction between the sources but instead limits the explanation to which location is active at any time point. The normal-mode approach, however, lays emphasis on the ERF as an emergent property of the systems dynamics of the entire auditory cortex. In this normal-mode view, adaptation is the result of a complete reorganisation of AC dynamics rather than of a reduction of activity in discrete sources. From this point of view, the spatially distributed normal mode is an even more fundamental building block of the ERF than the individual source.

The aim of the current study is to understand adaptation of the ERF. Building on our previous work presented in Hajizadeh et al. (2019), May and Tiitinen (2013), and May et al. (2015), we focus on this issue by extending normal mode analysis when the stimulus is repeated. To this end, we first provide general solutions to the state equations of the model, without the constraints that were necessary in our previous study (Hajizadeh et al. 2019). This allows us to reintroduce short-term synaptic depression into the model and to probe its adaptation behaviour when stimuli are presented at different repetition rates. Comparisons of model simulations with experimental MEG data are made. We go beyond describing adaptation of ERFs merely as an attenuation of the ERF response amplitude. Instead, we describe how the normal modes of the network dynamics, that is, the dynamics of the entire auditory cortex, changes as a result of stimulus repetition. Further, we investigate how adaptation lifetime depends on other factors than the dynamics of synaptic depression, namely gross anatomical structure and the balance between excitation and inhibition.

## Unfurling the model of auditory cortex

### Model description

We start with the model of AC, developed by May and colleagues (May and Tiitinen 2010, 2013; May et al. 2015; Westö et al. 2016). The model is based on the anatomical core-belt-parabelt organisation of AC. This coarse structure of auditory areas is similar across mammals, although species strongly differ in the number of auditory fields per area and the connectivity between fields (Kaas and Hackett 2000). The dynamics of the model were inspired by the work of Wilson and Cowan (1972) and Hopfield and Tank (1986). Its basic unit is a simplified description of the cortical column and comprises a mean-field excitatory and a mean-field inhibitory cell population that are characterised by the state variables $\varvec{u}(t)=\left( u_1(t),\dots ,u_N(t)\right) ^\top$ and $\varvec{v}(t)=\left( v_1(t),\dots ,v_N(t)\right) ^\top$, respectively, where *N* is the number of columns. Moreover, the dynamics of the excitatory state variables is also coupled with the synaptic efficacy $\varvec{q}(t)=\left( q_1(t),\dots ,q_N(t)\right) ^\top$. The dynamics of the model are then governed by the following set of coupled first-order differential equations (May and Tiitinen 2013; May et al. 2015; Hajizadeh et al. 2019)

1 $$\begin{aligned} \tau _{\mathrm{m}}\dot{\varvec{u}}(t)= & {} -\varvec{u}(t) + W_{\mathrm{ee}}Q(t) \cdot g[\varvec{u}(t)] - W_{\mathrm{ei}}\cdot g[\varvec{v}(t)] + \varvec{i}_{\mathrm{aff,e}}(t), \nonumber \\ \end{aligned}$$

2 $$\begin{aligned} \tau _{\mathrm{m}}\dot{\varvec{v}} (t)= & {} -\varvec{v}(t) + W_{\mathrm{ie}} \cdot g[\varvec{u}(t)] - W_{\mathrm{ii}}\cdot g[\varvec{v}(t)] + \varvec{i}_{\mathrm{aff,i}}(t), \end{aligned}$$

3 $$\begin{aligned} \varvec{\dot{q}}(t)= & {} -\frac{\varvec{q}(t)g[\varvec{u}(t)]}{\tau _{\mathrm{o}}} + \frac{\varvec{1}-\varvec{q}(t)}{{\tau _{\mathrm{rec}}}}, Q(t) = \hbox {diag}(\varvec{q}(t)). \end{aligned}$$

Here, $\tau _{\mathrm{m}}$ is the membrane time constant. The connections between excitatory (e) and inhibitory (i) cell populations are organised according to the anatomical structure of auditory cortex (Kaas and Hackett 2000) and are expressed by the four weight matrices $W_{\mathrm{ee}}$, $W_{\mathrm{ei}}$, $W_{\mathrm{ie}}$, and $W_{\mathrm{ii}}$. The elements of the matrices $W_{\mathrm{ee}}$ and $W_{\mathrm{ie}}$ describe excitatory-to-excitatory and excitatory-to-inhibitory connections, respectively, and encompass all the connections *between* the columns. Note that only $W_{\mathrm{ee}}$ includes long-range connections between areas, and $W_{\mathrm{ie}}$ describes lateral inhibition. $W_{\mathrm{ei}}$ and $W_{\mathrm{ii}}$ comprise local inhibitory-to-excitatory and inhibitory-to-inhibitory connections, which only occur *within* a column, and these matrices are, thus, diagonal. The firing rates $g[\varvec{u}(t)]$ and $g[\varvec{v}(t)]$ are component-wise sigmoid functions of the form $g[\varvec{x}]=\tanh [\alpha \varvec{x}]$ where $\varvec{x}$ stands for $\varvec{u}(t)$ or $\varvec{v}(t)$. The parameter $\alpha$ is a scalar which determines the sensitivity of the firing rate to the value of the respective state variable. The variables $\varvec{i}_{\mathrm{aff,e}}(t)$ and $\varvec{i}_{\mathrm{aff,i}}(t)$ are time-dependent subcortical afferent inputs. Equation (1) indicates that the excitatory-to-excitatory connections are not static and are modulated by short-term synaptic depression, which is defined as $\varvec{d}(t) = \varvec{1}-\varvec{q}(t)$. This is expressed by the matrix multiplication of the elements of $W_{\mathrm{ee}}$ with the synaptic efficacy *Q*(*t*), which is a time-dependent diagonal matrix. Further, $\tau _{\mathrm{o}}$ and $\tau _{\mathrm{rec}}$ are the time constants of the release and the replenishment of neurotransmitters at each synapse; and $\varvec{1}$ is the 1-vector of size *N*. Note that $\varvec{q}(t)$ is also a vector and the multiplication between the vectors is a component-wise operation. Equation (3) implies that the synaptic strength between pre- and post-synaptic cell populations depends only on the activity of the state variable $\varvec{u}(t)$ of the pre-synaptic excitatory cell population. Inclusion of synaptic plasticity as it is given in Eq. (3) in the model was inspired by Tsodyks and Markram (1997) and Loebel et al. (2007). With known connectivity matrices $W_{\mathrm{ee}}$, $W_{\mathrm{ei}}$, $W_{\mathrm{ie}}$, and $W_{\mathrm{ii}}$ as well as the input terms $\varvec{i}_{\mathrm{aff,e}}(t)$ and $\varvec{i}_{\mathrm{aff,i}}(t)$, the nonlinear system described by Eqs. (1)–(3) can be solved numerically to provide a picture of the spatiotemporal activity of AC.

There is a richness of dynamical behaviour that the model can display. This is because the diagonal entries of the connectivity matrices determine the oscillator properties of each column. For values used in our parametrisation (see Table 1), any single column without coupling and input behaves as a damped oscillator. For larger values on the diagonal elements of $W_{\mathrm{ee}}$ and $W_{\mathrm{ei}}$, self-sustained oscillations can appear. For the case of damped oscillators, we may use linear firing rates $g[x]= \alpha x$ such that Eqs. (1) and (2) with fixed synaptic efficacy $\varvec{q}(t) = \varvec{q}$ behave essentially as a network of coupled linear filters. However, when synaptic efficacy is a dynamical variable due to STSD, the full model represented in Eqs. (1), (2), and (3) remains nonlinear through the $Q(t)\varvec{u}(t)$ terms in Eqs. (1) and (3).

### Solution by normal modes

Hajizadeh et al. (2019) demonstrated that, under certain assumptions, the solutions for Eqs. (1) and (2) can be written as a linear combination of normal modes. These assumptions are that the firing rate is linear ($g[x] = \alpha x$) ( see also, Allen et al. 1975; Katznelson 1981; May and Tiitinen 2001), synaptic efficacy is constant, i.e., $Q(t)\equiv I$, and the connection matrices are symmetric. Hajizadeh et al. (2019), then, realized eigenvalue decomposition by first transforming Eqs. (1) and (2) into second-order differential equations which refer to the oscillating nature of brain activity.

In contrast to the approach of Hajizadeh et al. (2019), we strive here for general solutions of Eqs.  (1) and (2) by including the dynamics of STSD and without a diversion via a system of second-order differential equations. To this end, the homogeneous part of Eqs. (1) and (2) is rewritten in the form of a standard linear system

4 $$\begin{aligned} \begin{aligned}&\!\!\!\begin{pmatrix} \dot{\varvec{u}}(t) \\ \dot{\varvec{v}}(t) \end{pmatrix} = M \begin{pmatrix} \varvec{u}(t) \\ \varvec{v}(t) \end{pmatrix} \qquad \text {with}\qquad \\&M= \frac{1}{\tau _{\mathrm{m}}} \begin{pmatrix} \alpha {W}_{\mathrm{ee}}Q -I&{} -\alpha {W}_{\mathrm{ei}} \\ \alpha {W}_{\mathrm{ie}} &{} -\alpha {W}_{\mathrm{ii}}-I \end{pmatrix}, \end{aligned} \end{aligned}$$

where *I* is the identity matrix. The general solution to Eq. (4) is then given by linear combinations

5 $$\begin{aligned} \begin{pmatrix} \varvec{u}(t) \\ \varvec{v}(t) \end{pmatrix} = \displaystyle \sum _{n=1}^{2N} c_n \hbox {exp}(\lambda _n t) \begin{pmatrix} \varvec{x}_n \\ \varvec{y}_n \end{pmatrix} , \end{aligned}$$

where $\lambda _n\in {\mathbb {C}}$, $n=1,\dots ,2N$ are the eigenvalues of the coefficient matrix *M* in Eq. (4). The eigenvectors $\left( \varvec{x}_n,\varvec{y}_n\right) ^\top$ are the normal modes, where $\varvec{x}_n$ and $\varvec{y}_n$ represent the collection of the $\varvec{u}$ and $\varvec{v}$ components of the *n*-th eigenvector. For a specific initial condition $\left( \varvec{u}(0),\varvec{v}(0)\right) ^\top =\left( \varvec{u}_0,\varvec{v}_0 \right) ^\top$, the complex coefficients $c_n$ are given as scalar products

6 $$\begin{aligned} c_n=\left< \begin{pmatrix} \varvec{u}_0\\ \varvec{v}_0 \end{pmatrix}, \begin{pmatrix} \varvec{\xi }_n\\ \varvec{\eta }_n \end{pmatrix} \right>, \end{aligned}$$

with the corresponding left eigenvectors $\left( \varvec{\xi }_n,\varvec{\eta }_n\right) ^\top$ of the coefficient matrix *M*. For all reasonable choices of the weight matrices in Eq. (4), the matrix *M* is stable, that is, all the eigenvalues $\lambda _n=\gamma _n+i\omega _n$ for a given angular frequency $\omega _n$ have a decay rate $\gamma _n<0$. If $\omega _n\ne 0$, the normal mode dynamics are of the underdamped type and, thus, the eigenvalues and their corresponding eigenvectors appear in complex conjugate pairs. For real initial values $\left( \varvec{u}(0),\varvec{v}(0)\right) ^\top$, the corresponding pair of complex coefficients $c_n$ has to be complex conjugate as well. The modulus of the complex coefficient $c_n$ is the initial amplitude of the mode whilst its argument provides the initial phase. If $\omega _n=0$, the normal modes are of the overdamped type, and the eigenvectors together with their coefficients are real.

### Dynamics of STSD and the slow-fast approximation

Here, we study adaptation dynamics in AC using a paradigm where the AC is excited by a sequence of tones periodically delivered *S* times with an identical stimulus onset interval between two consecutive stimuli. With repetitive stimulation, the system responds most strongly to the first stimulus; we refer to this condition as the *initial state*. Within the next few stimuli, STSD increases and, therefore, the response magnitude rapidly decreases and finally approaches a constant value. We call this state of the system the *adapted state*, where further incoming stimuli induce only small changes in the response. The adapted state is described by a balance between fast depression and recovery from depression—governed by the time constants in Eq. (3)—and strongly depends on the temporal pattern of the stimulation. Without any further stimulation, the system recovers back to its initial state with the time constant $\tau _{\mathrm{rec}}$, which is much larger than $\tau _{\mathrm{o}}$.

Assuming that the stimulus duration is short compared to the time scales of the system, we can include the stimuli in our model as input functions of the form

7 $$\begin{aligned} \varvec{i}_{\mathrm{aff,e}}(t)=\varvec{a}\sum _{s=0}^{S}\delta (t-t_s), \end{aligned}$$

where the *s*-th stimulus appears at $t_s=s\cdot \hbox {SOI}$, and the vector $\varvec{a}$ gives the input strengths at each column in the network. Here, only the first element of $\varvec{a}$ is non-zero. That is, the afferent input occurs only and specifically in the excitatory cell population of IC, i.e., $\varvec{i}_{\mathrm{aff,i}}(t)= 0$. From IC the signal propagates to the AC via thalamus. Note that, in principle, the model is able to deal with any type of input function. However, describing the stimuli as delta functions allows us to treat the impact of the stimuli as jumps of $\varvec{u}(t)$ and $\varvec{v}(t)$ at the stimulation times $t_s$, while in the time intervals between the stimuli, we can use the homogeneous Eq. (4). Together with further slight simplifications of the model, which we describe below, this will enable us to perform a stimulus-wise normal mode analysis of the system as it adapts to repetitive stimulation.

Since $\tau _{\mathrm{o}} \ll \tau _{\mathrm{rec}}$, the dynamics of $\varvec{q}(t)$, given in Eq. (3), is characterised by two different time scales: First, there is a fast drop-off $(-1/\tau _{\mathrm{o}})(\varvec{q}(t)g[\varvec{u}(t)])$ occurring directly after a stimulus when the firing rate $g[\varvec{u}(t)]$ is non-zero. Second, there is a slow recovery phase when the firing rates $g[\varvec{u}(t)]$ have decayed and Eq. (3) is governed by the recovery term $(1/{\tau _{\mathrm{rec}}})(\varvec{1}-\varvec{q}(t))$. Following the general mathematical theory for slow-fast systems (see, for example, Kuehn 2015), we can use this time-scale separation to introduce a slow-fast approximation of the STSD process. We keep the synaptic efficacy at a constant value $Q(t)=Q_s$ in each time interval $t\in [t_s,t_{s+1}]$ between two consecutive stimuli and update it together with the stimulus-induced jumps of $\varvec{u}(t)$ and $\varvec{v}(t)$ at the stimulation times $t_s$.

For the updating $Q_s\mapsto Q_{s+1}$, we separate the processes of fast drop-off during the stimulus-induced activity from the slow recovery after the stimulus-induced activity. The fast drop-off ${\mathcal {F}}_s(\varvec{q}_s)$ is obtained by integrating the first term in Eq. (3)

8 $$\begin{aligned} \small {{\mathcal {F}}_s(\varvec{q}_s) = \varvec{q}_s \hbox {exp}\left( {-\frac{1}{\tau _{\mathrm{o}}}\int _{t_s}^{t_{s+1}} g[\varvec{u}(t^\prime )] dt^\prime }\right) ,} \end{aligned}$$

whereby we treat neurotransmitter release as a process independent of vesicle replenishment. Inserting this as initial value into the slow recovery process, which can be explicitly integrated, we obtain the combined update as given by

9 $$\begin{aligned} \varvec{q}_{s+1} = \varvec{1}- \left( \varvec{1}-{\mathcal {F}}_s(\varvec{q}_s)\right) \hbox {exp}\left( -{\frac{t_{s+1}-t_s}{\tau _{\mathrm{rec}}}}\right) . \end{aligned}$$

Inserting the general solution from Eq. (5) in Eq. (8), ${\mathcal {F}}_s(\varvec{q}_s)$ can be rewritten as

10 $$\begin{aligned} {\mathcal {F}}_s(\varvec{q}_s)= \varvec{q}_s \prod \limits _{n=1}^{2N} \hbox {exp}\left( \frac{-c_{n,s} \left( \hbox {exp}\left( \lambda _{n,s} \left( t_{s+1}-t_s\right) \right) - 1\right) }{ \tau _{\mathrm{o}}\lambda _{n,s}} \varvec{x}_{n,s}\right) .\nonumber \\ \end{aligned}$$

Note that for each time interval $[t_s,t_{s+1}]$, we have to use the step-wise adapting coefficient matrix $M_s=M(Q_s)$ to recalculate the normal modes $\left( \varvec{x}_{n,s},\varvec{y}_{n,s}\right) ^\top$, the eigenvalues $\lambda _{n,s}$, and the coefficients $c_{n,s}$ for which we also need the left eigenvectors $\left( \varvec{\xi }_{n,s},\varvec{\eta }_{n,s}\right) ^\top$. Further, we assume that between two consecutive stimuli the state variables $\varvec{u}(t)$ and $\varvec{v}(t)$ have decayed to zero so that the next stimulus induces an abrupt increase of $\varvec{u}(t)$ and $\varvec{v}(t)$. This means that at each time point $t_s$, based on the stimulus history, the dynamics of Eq. (4) are reparameterised by updating $Q_s$, and $\varvec{u}(t)$ and $\varvec{v}(t)$ are set to a new stimulus-induced starting value. In particular, although the input $\varvec{a}\delta (t-t_s)$ is the same at the beginning of each interval, the effective input to the normal modes differs. It is determined by the adapting connectivity pattern of the network, which itself depends on the stimulus history by means of STSD. In this way, the slow-fast approximation allows for stimulus-wise normal mode analysis of Eq. (4) in each time interval $t\in [t_s,t_{s+1}]$ between two consecutive stimuli, where the synaptic efficacy variables $Q(t)=Q_s$ stay piecewise constant. We will use this later as a tool for analysing the STSD induced changes in the generation of the ERF signals.

**Fig. 1:**
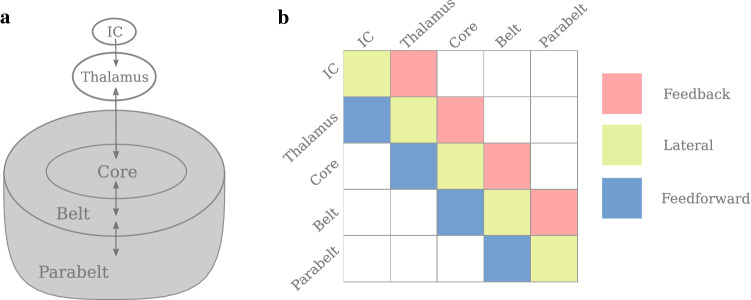
Schematic representation of the anatomical structure of the model. **a** The structure is divided into subcortical and cortical regions. The subcortical areas IC and thalamus provide the afferent input to the AC. The AC consists of the serially organised core, belt, and parabelt areas. There are therefore a total of five areas, and each area is represented by a population of excitatory and a population of inhibitory neurons; each population is described by a single state variable. Additionally, the connections from each excitatory population are modulated by STSD. Thus, the model is a 15-dimensional system of coupled first-order differential equations. **b** The structure is represented in the connection matrix $W_{\mathrm{ee}}$ with non-zero matrix elements $w^\text {(ff)}_{\mathrm{ee}}$ (feedforward, blue), $w^\text {(fb)}_{\mathrm{ee}}$ (feedback, red), and $w^\text {(d)}_{\mathrm{ee}}$ (lateral, yellow). Note that the other three weight matrices, not shown here, are diagonal square matrices of order five

**Fig. 2:**
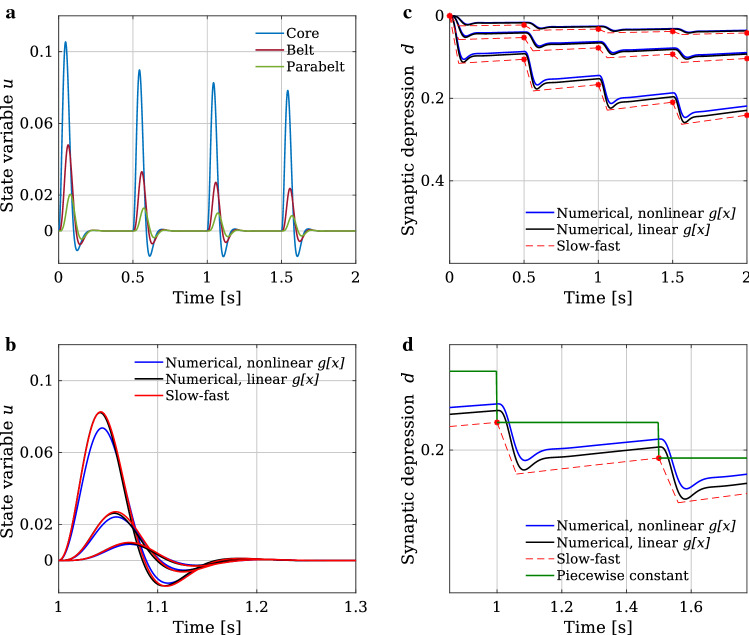
State variable *u*(*t*) and STSD *d*(*t*) derived with the slow-fast approximation compared with the numerical simulations of the full model given in Eqs. (1)–(3). **a** The state variable *u*(*t*) as a response to a sequence of identical stimuli with constant SOI of 0.5 s is shown for core (blue), belt (red), and parabelt (green) areas. For each area, the first stimulus generates the largest response. Although the state variables decay to zero during the interval between two consecutive stimuli, the excitatory-to-excitatory connections are weakened by STSD and this effect carries over the silent period of the SOI so that the peak amplitude of *u*(*t*) decreases until it levels off after a few stimuli. **b** The state variable *u*(*t*) as a response to one stimulus computed with the slow-fast approximation (red) is contrasted with that gained in numerical simulations using nonlinear firing rates $g[x] = \tanh (\alpha x)$ (blue) and linear firing rates $g[x] = \alpha x$ (black). The solutions based on the slow-fast approximation provide a good estimation of numerical solutions. The high-, intermediate-, and low-amplitude responses are from the core, belt, and parabelt, respectively. **c** The STSD time course estimated with the slow-fast approximation (red) agrees well with the numerical simulations with nonlinear (blue) and linear (black) firing rates. The red dashed lines represent solutions to Eq. (9), and the red dots indicate the onset of stimulus presentation, at which the fast drop-off according to Eq. (8) is computed. **d** This plot is an enlarged version of the STSD variable of the corresponding time interval shown in (**b**). In order to compute the state variables using the slow-fast approximation, we assumed that STSD is piecewise constant in the time interval between the onsets of two consecutive stimuli as indicated by the green horizontal line. The corresponding STSD value at each stimulus onset (red dots) was derived from the slow-fast approximation given in Eqs. (8) and (9)

### An auditory cortex model with a simplified structure

For the simulations presented in this work, we used a model with a strongly simplified anatomical structure that encompassed two subcortical areas, viz. IC and thalamus, and three cortical areas representing the core, the belt, and the parabelt (see Fig. 1a). For reasons of simplicity, each of the five areas consisted of only one auditory field, which, in turn, comprised just one column with an excitatory and an inhibitory cell population. A central feature of this network is its serial connectivity, i.e., only neighbouring areas are directly connected with each other via feedforward and feedback connections, as illustrated for the connection matrix $W_{\mathrm{ee}}$ in Fig. 1b. The only type of connection *between* two columns are excitatory-to-excitatory connections. All other connections are assumed to be local and existing within a single area. Therefore, their corresponding connection matrices are diagonal and of the order five given by $W_{\mathrm{ie}}=w^\text {(d)}_{\mathrm{ie}}{I}$, $W_{\mathrm{ei}}=w^\text {(d)}_{\mathrm{ei}}{I}$, and $W_{\mathrm{ii}}=w^\text {(d)}_{\mathrm{ii}}{I}$.

Figure 2a shows an example of the model output in terms of the state variable $\varvec{u}(t)$ based on the slow-fast approximation for a repeated stimulation of the network with SOI = 0.5 s. The parameters used for the computation are summarised in Table 1. For each stimulus, the peak magnitudes of $\varvec{u}(t)$ gradually decrease—and the corresponding peak latencies increase—as one moves up the hierarchy from the core to the parabelt. The excitatory-to-excitatory connections of $W_{\mathrm{ee}}$ weaken due to STSD (i.e., due to a lowering of synaptic efficacy *Q* in Eq. (4)). Consequently, in each area, the peak amplitude of $\varvec{u}(t)$ decreases across stimulus presentation.

**Table 1:** Default parameter values used for the simulations. These values were chosen to replicate realistic-looking ERFs

Dynamical parameters	Value	Topographical parameters	Value
$w^\text {(d)}_{\mathrm{ee}}$	2	$k^\text {(d)}_1$	−1
$w^\text {(ff)}_{\mathrm{ee}}$	0.5	$k^\text {(ff)}_1$	−1
$w^\text {(fb)}_{\mathrm{ee}}$	0.4	$k^\text {(fb)}_1$	15
$w^\text {(d)}_{\mathrm{ie}}$	3.5	$k^\text {(d)}_2$	2
$w^\text {(d)}_{\mathrm{ei}}$	2.2		
$w^\text {(d)}_{\mathrm{ii}}$	2.5		
$\tau _{\mathrm{m}}$	0.03 s		
$\tau _{\mathrm{o}}$	0.04 s		
$\tau _{\mathrm{rec}}$	5 s		
*a*	0.02		
$\alpha$	1		

Figure 2b shows the state variable $\varvec{u}(t)$ from the numerical simulations with nonlinear (blue) and linear (black) firing rates, and from the slow-fast approximation (red). All simulations were computed using the same set of model parameters displayed in Table 1 and the identical input function given in Eq. (7). There is a close correspondence between the simulations: the simplifications induced by linear firing rates and the slow-fast approximation have no relevant impact on the waveforms and their adaptation. The evolution of the corresponding STSD variables $\varvec{d}(t) = \varvec{1}-\varvec{q}(t)$ is shown in Fig. 2c. Again, there are only minor discrepancies between the nonlinear (blue) and linear (black) solutions. For the slow-fast approximation, the STSD variables are updated only at the stimulation times $t_s$ (red points). Figure 2d illustrates the operation of the slow-fast approximation where, in Eq. (4), the evolution of $\varvec{u}(t)$ and $\varvec{v}(t)$ is computed using the piecewise constant values (green) of the STSD process. Note that, as explained in the Sect. 2.3, the combination of the approximations for the fast drop (Eq. (8)) and the slow recovery (Eq. (9)) (red dashed) are only used to obtain the values of $\varvec{q}_s$ which stay piecewise constant during the evolution of $\varvec{u}(t)$ and $\varvec{v}(t)$. Note also that the lemniscal subdivisions of IC and thalamus have much faster recovery time constants compared to the cortical regions (Asari and Zador 2009; Pérez-González and Malmierca 2014; Ulanovsky et al. 2004). Therefore, in our simulations, IC and thalamus do not adapt, i.e., the corresponding values of synaptic efficacy *Q*(*t*) for the two subcortical areas are set to unity in all simulations. Figure 2 demonstrates that the slow-fast approximation, described in Sect. 2.3, provides good estimations of the numerical simulations of Eqs. (1), (2), and (3). Therefore, it can be used in lieu of numerical solutions to study the adaptation dynamics of AC.

### Computing MEG signals

MEG signals are generated mainly by primary currents running in the apical dendrites of synchronously active pyramidal cells in the cortex (Hämäläinen et al. 1993). The apical dendrites are locally aligned with each other and point in a direction perpendicular to the cortical surface. When a portion of the cortex becomes active, its contribution to the MEG signal is proportional to the total current running in the apical dendrites. This is weighted by the distance to the MEG sensor and by the orientation of the current, which is determined by the local gyrification of the cortical surface. The primary current in each apical dendrite is driven by the synaptic inputs to the dendrite. This means that each synaptic input contributes to the MEG signal, and the magnitude and polarity of this contribution depends on the location of the synapse on the dendritic tree and on the type of the synapse (Ahlfors and Wreh 2015). An excitatory synapse near the cell body will cause a positive current to be pumped up the tree, towards the cortical surface. Conversely, an excitatory synapse near the distal end of the tree will cause the current to travel in the opposite direction, away from the cortical surface. Consequently, feedforward connections, which generally target the proximal dendrites in layer IV, result in a current pointing towards the cortical surface. In contrast, feedback input arriving in the upper layers produce a current pointing downward (Ahlfors et al. 2015).

**Fig. 3:**
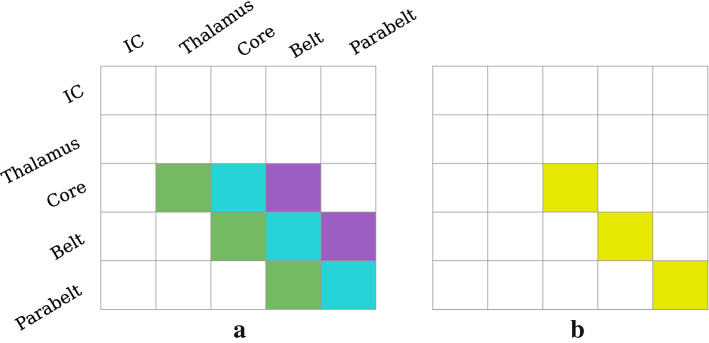
The $K_1$ (**a**) and $K_2$ (**b**) matrices, which contain the information about the topology of the primary currents, provide connection-specific multipliers of $W_{\mathrm{ee}}$ and $W_{\mathrm{ei}}$ in the computation of the MEG signal, respectively. **a** The green ($k^\text {(ff)}_1$) and purple ($k^\text {(fb)}_1$) elements in $K_1$ represent feedforward and feedback connections, respectively. The cyan elements ($k^\text {(d)}_1$) on the diagonal represent the lateral connections. Contributions of IC and thalamus to the MEG are zero, but the excitatory connections from the thalamus to the core contribute to the MEG. **b** The yellow elements ($k^\text {(d)}_2$) in $K_2$ represent the weights of inhibitory connections

We modelled MEG generation with the above considerations in mind. Given that the MEG signal of a pyramidal cell is quite well approximated by the synaptic input current of the neuron (May 2002), the MEG contribution from each area is assumed to be proportional to the input to the column representing the area. These inputs are defined by the second and third term on the right hand side of Eq. (1). Each input is weighted by a connection-specific multiplier which depends on the connection type (feedforward, feedback, excitatory, inhibitory) (for more information, see Hajizadeh et al. 2019, 2021). This topological information is expressed in the matrices $K_1$ and $K_2$, whose structures are shown in Fig. 3. They specify how each synaptic connection contributes to the MEG signal by an element-wise multiplication (Hadamard product $\circ$) with the matrices $W_{\mathrm{ee}}$ and $W_{\mathrm{ei}}$. Thus, the total MEG signal is the product of $K_1$ and $K_2$ representing the topography, the synaptic strengths represented in $W_{\mathrm{ee}}$ and $W_{\mathrm{ei}}$, and the firing rate of the pre-synaptic cell population. Therefore, the MEG signal *R*(*t*) is computed as

11 $$\begin{aligned} R(t) = \sum _{j=1}^{2N} \left[ \begin{pmatrix} K_1\circ W_{\mathrm{ee}} &{} O \\ O &{} K_2\circ W_{\mathrm{ei}} \end{pmatrix} \begin{pmatrix} \varvec{u}(t) \\ \varvec{v}(t) \end{pmatrix}\right] _j, \end{aligned}$$

where *j* runs over the number of cortical columns in the model, and *O* is the zero matrix of order *N*.

**Fig. 4:**
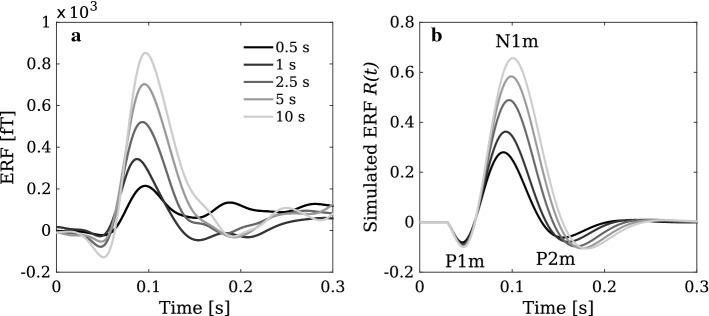
Comparison of experimental and simulated ERFs showing the dependence of the ERF response on SOI. **a** Trial-averaged ERF responses from an MEG sensor in the vicinity of auditory cortex recorded from a single subject. The peak amplitude of the N1m shows a high sensitivity to changes in SOI (data from Zacharias et al. (2012)). **b** Simulated ERF responses calculated by use of the slow-fast approximation. These replicate all the major landmarks of the dependence of the experimental ERF on SOI: the P1m as well as the rising slope of the N1m are least affected by SOI. As SOI is increased, the peak latency of the N1m grows and the falling slope of the N1m becomes steeper. We have added a 30-ms shift to the simulated waveforms to account for the time delay due to sub-cortical processing

In Fig. 4, we compare experimental MEG data with simulations based on the slow-fast approximation using Eq. (11). MEG signals were recorded from a single subject who passively listened to sequences of tones. These tones (audio frequency 1.5 kHz, duration 100 ms, sound-pressure level 80 dB) were presented in five different blocks (111 tone repetitions per block) with constant SOI between two consecutive tones. The SOIs of the blocks were 0.5 s, 1 s, 2.5 s, 5 s, and 10 s, corresponding to stimulation rates of 2 Hz, 1 Hz, 0.4 Hz, 0.2 Hz, and 0.1 Hz, respectively (Zacharias et al. 2012). Figure 4a shows the trial-averaged waveforms for the five SOIs. With increasing SOI, the N1m peak magnitude and the corresponding peak latency (except for the 0.5-s SOI) increases, thus presumably reflecting the different speed of recovery from STSD for short and long SOIs. Furthermore, the rising slope of the N1m is unaffected by the SOI, whereas the falling slope clearly differs between the five waveforms. Also, P2m seems to be more affected by the adaptation process than the P1m deflection.

Using the same stimulation paradigm as described for the experimental data, we performed simulations of ERFs based on the normal mode analysis (Eq. (11)) with the slow-fast approximation and using the parameters given in Table 1. As shown in Fig. 4b, the simulations replicate the main characteristics of the experimental data (Fig. 4a): (1) As SOI is increased, both the peak amplitude and the peak latency of the N1m become larger, (2) the rising slope of the N1m is unaffected by SOI, (3) the falling slope of the N1m becomes systematically steeper as SOI grows, and (4) the width of the N1m waveform increases as a function SOI. With the use of the slow-fast approximation we have now a tool at hand that enables us to investigate how stimulus repetition modifies the dynamics of AC.

## Modelling results: novel views on ERF adaptation

In this section, we first show how normal modes in time and space contribute to the formation of ERFs and to their adaptation with stimulus repetition. Second, we show how ERF adaptation can be viewed from the perspective of different physiological connections. Third, we demonstrate that the recovery from adaptation depends not only on the system parameters, such as time constants which directly regulate the STSD dynamics. Rather, adaptation reflects also the network structure and varies from area to area in terms of recovery time. Finally, we use our model to quantify the adaptation of the N1m for smaller and larger SOIs and in this way discuss the limitations of the widely-used single exponential function as a description of adaptation recovery.

**Fig. 5:**
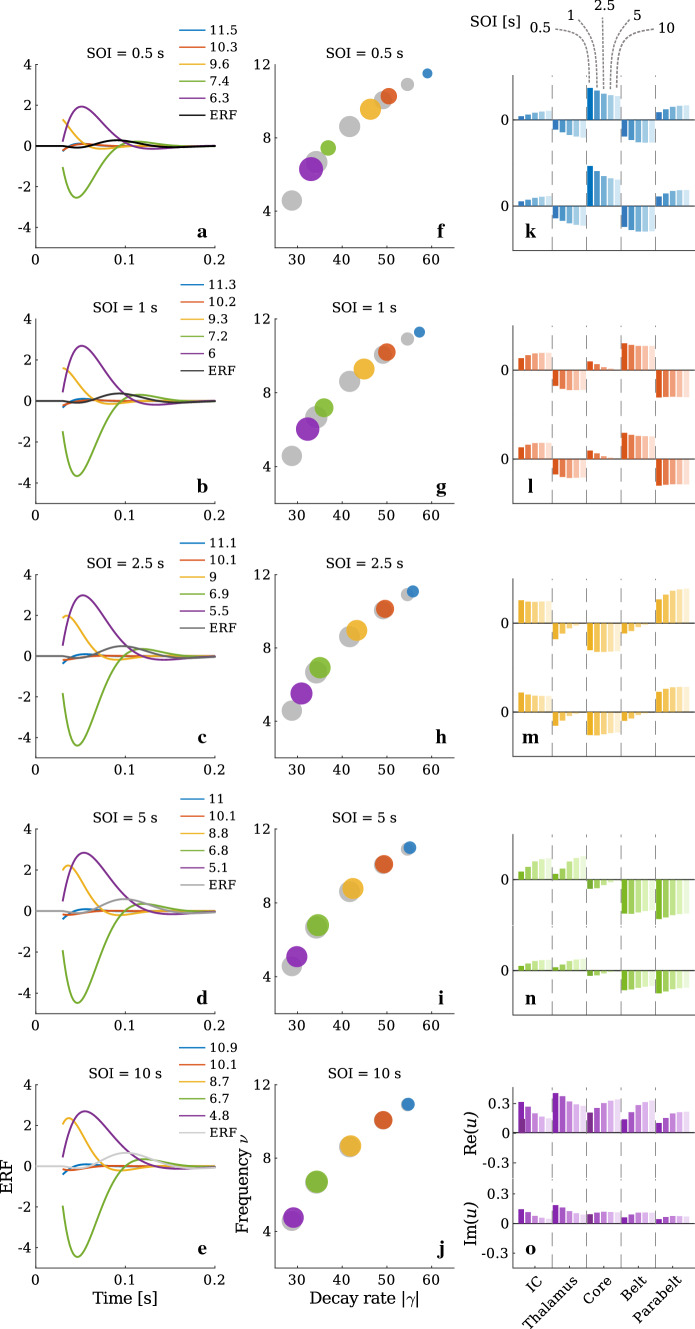
Decomposition of the simulated ERFs into normal modes. **a–e** Each ERF (shaded grey) is displayed together with the underlying normal modes, represented by different colours which stay consistent in the left, middle, and right columns of this figure. The ERFs are the same as those presented in Fig. 4b. For each SOI, all normal modes peak earlier than the corresponding N1m, and the main contributor to the ERFs are the low-frequency normal modes (purple and green) appearing in opposite phase. The high-frequency normal modes (blue and red) decay fast and contribute only very weakly to the N1m and P2m responses. **f–j** Dispersion relation between frequency (Im$(\lambda )/{2\pi }=\nu )$ and the absolute value of the decay rate (|Re$(\lambda )|=|\gamma |$). The spectral information shows that all normal modes are of the underdamped type. The grey discs represent the dispersion relation at the initial state, which is the same for all SOIs. The coloured discs correspond to the dispersion relation of the adapted state. In general, frequency and decay rate of the normal modes increase with decreasing SOI. The size of the discs are proportional to the initial amplitude $|c_n|$ of the normal modes. In both the initial state and the adapted state, the low-frequency normal modes have a larger amplitude than the high-frequency normal modes. **k**–**o** The spatial wave patterns for different modes in different SOIs are given in the eigenvectors $\left( \varvec{x}_n,\varvec{y}_n\right) ^\top$. Here, the real and the imaginary parts of the excitatory state variables $\varvec{u}(t)$ are shown, which, for each mode individually, follow the same pattern. As indicated in panel **k**, the different shades signify different SOIs. The high-frequency modes **k–l** occur with large spatial wave number, whereas the low-frequency modes **m–o** appear with low wave number in space. Moreover, the same spatial wave pattern is observed for all SOIs

### Adaptation of ERFs as a result of adapting normal modes

According to Eq. (11), the MEG signal *R*(*t*) is a function of the excitatory and inhibitory state variables $\varvec{u}(t)$ and $\varvec{v}(t)$. By a change of coordinate, *R*(*t*) can be expressed in terms of normal modes so that the only time-varying term is the normal mode amplitude, whereas the oscillation frequency and damping rate remain constant. Substituting Eq. (5) in Eq. (11) results in

12 $$\begin{aligned} R(t) = \displaystyle \sum _{j=1}^{2N} \left[ \begin{pmatrix} K_1\circ W_{\mathrm{ee}} &{} O \\ O &{} K_2\circ W_{\mathrm{ei}} \end{pmatrix} \displaystyle \sum _{n=1}^{2N} c_n \hbox {exp}(\lambda _n t) \begin{pmatrix} \varvec{x}_n \\ \varvec{y}_n \end{pmatrix}\right] _j. \nonumber \\ \end{aligned}$$

The left column of Fig. 5 shows the simulated ERF waveforms and the underlying normal modes of the adapted states for the five different SOIs presented in Fig. 4b. The ERFs are displayed as grey curves, with the same shades of grey used in Fig. 4b. The normal modes are depicted in different colours; they are identifiable by their characteristic frequencies $\nu =\omega /2\pi$ in the legend. The ERF decomposition results in normal modes whose polarities are preserved across SOI. For all SOIs, each normal mode peaks well before the N1m, during the first 50 ms. The two normal modes with the lowest $\nu$ (purple and green curves) show by far the largest amplitude. They have opposite phases and a peak latency at around 50 ms. In contrast, the two modes with the highest frequencies (blue and red) have much smaller magnitudes and decay faster. The mode with the intermediate frequency (yellow) has a prominent peak magnitude with a small latency. As a consequence of this diversity of normal modes, the P1m of the ERFs is composed from all the modes, whereas the N1m and P2m are predominantly formed by the two modes with the lowest frequencies.

The middle column of Fig. 5 shows the dispersion relation of the normal modes underlying the ERFs. At both initial and adapted states, the normal modes are of the underdamped type because their frequencies $\nu$ are non-zero and their corresponding decay rates $\gamma$ are negative. Moreover, for all SOIs there is a typical common dispersion relation between frequency and decay rate, implying that modes with higher frequency also decay faster. Additionally, for all SOIs, the frequencies and the decay rates of the modes in the adapted state shift to larger values compared to the initial state; this shift is larger the smaller the SOI is. For the largest SOI, there are only minute differences between the dispersion relation at the initial and the adapted state, because the 10-s interval between two successive stimuli provides sufficient time for the synaptic efficacy *q*(*t*) to return to the initial state.

Stimulus history changes not only the frequency and the decay rate of the normal modes but also their initial amplitude $|c_n|$ after each stimulus (see Eq. (6)). These amplitudes are indicated by the size of each disc in Fig. 5f–j: the larger the size of a disc, the larger the initial amplitude of the respective normal mode. Further, Fig. 5 shows that, in the initial state (grey discs), there is a gradual decrease of the initial amplitude $|c_n|$ from low- to high-frequency modes, whereas in the adapted state there is no systematic pattern except that, for all stimulation rates, the normal mode with the highest frequency (blue discs) has the smallest initial amplitude.

Normal modes are not only oscillations in time but they manifest themselves as spatial wave patterns. This information is provided by the eigenvectors $\left( \varvec{x}_n,\varvec{y}_n\right) ^\top$ of the coefficient matrix *M*. The real and the imaginary parts for the state variables $\varvec{u}(t)$ for the five areas of the network are represented in the right column of Fig. 5, with the mode with the highest frequency $\nu$ on top and the one with the lowest frequency $\nu$ at the bottom of the column. As indicated in Fig. 5k, different shades of each colour stand for different SOIs. Note the strong similarity of the spatial wave patterns between the real and imaginary parts of $\varvec{u}(t)$ for all modes. For all SOIs and for both the real and imaginary parts of $\varvec{u}(t)$, the wave number in space decreases from the high-frequency mode (blue, Fig. 5k) to the low-frequency mode (purple, Fig. 5o). The same pattern is also observed for the state variable $\varvec{v}(t)$, which is not shown here.

*Input efficiency and MEG efficiency*

Figure 5 indicates that the normal modes contributing to the generation of the ERFs are all different from one another. According to Eq. (12), these different contributions do not only originate from the differences in the initial amplitudes. Reformulating Eq. (12) as

13 $$\begin{aligned} \begin{aligned}&R(t) = \displaystyle \sum _{n=1}^{2N} c_n \kappa _n\hbox {exp}(\lambda _n t) \qquad \text {with}\qquad \\&\kappa _n= \sum _{j=1}^{2N} \left[ \begin{pmatrix} K_1\circ W_{\mathrm{ee}} &{} O \\ O &{} K_2\circ W_{\mathrm{ei}} \end{pmatrix} \begin{pmatrix} \varvec{x}_n \\ \varvec{y}_n \end{pmatrix}\right] _j, \end{aligned} \end{aligned}$$

we see that the contribution of each normal mode to the MEG signal is proportional to two factors:

1. the initial amplitude $|c_n|$ of the mode, interpreted as the *input efficiency* with respect to the stimulation pattern, and
2. the *MEG efficiency* $\kappa _n$, describing to which extent the MEG device is able to detect the mode.

**Fig. 6:**
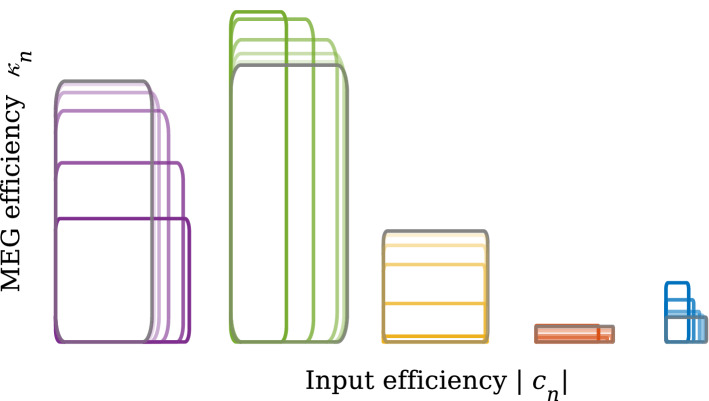
Characterisation of normal modes in terms of MEG efficiency $\kappa _n$ and input efficiency $|c_n|$. The normal modes are clustered according to the five frequency bands and they are colour-coded as in Fig. 5. Frequency increases from left to right. Within each band, each normal mode is represented by a rectangle, whose width ($\Delta$x) and height ($\Delta$y) is equivalent to $|c_n|$ and $\kappa _n$, respectively. SOI is represented by the colour shade, with dark shades indicating the smallest and light shades the largest SOI. The initial state is in grey. For all SOIs, the two low-frequency modes (purple and green) contribute most to the ERFs, whereas the contributions of the two high-frequency modes (red and blue) are negligible. Input efficiency shows less variability than MEG efficiency, with the exception of the normal mode with the second-lowest frequency (green)

This information is illustrated in Fig. 6, where the input efficiency $|c_n|$ and MEG efficiency $\kappa _n$ of each normal mode in the initial (grey) and adapted (coloured) states are characterised by a rectangle of width $|c_n|$ and height $\kappa _n$. The area of each rectangle is proportional to the absolute value of the total contribution of a given normal mode to the ERFs presented in Fig. 4b. This figure shows that the two low-frequency modes (purple and green) are the major contributors to the MEG signals. As SOI decreases, the MEG efficiency of the lowest frequency normal mode (purple) decreases, but its input efficiency increases. In contrast, the contribution of the second mode (green) increases in MEG efficiency and decreases in input efficiency. The input efficiency of the third mode (yellow) is relatively unaffected by SOI, whereas its MEG efficiency decreases considerably for smaller SOIs. The total contribution of the two high-frequency modes is negligibly small.

**Fig. 7:**
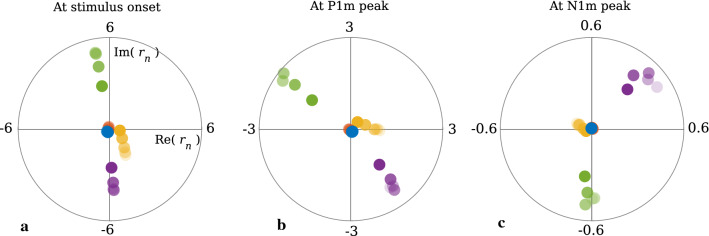
Normal modes $r_n$ underlying the ERF presented in the complex plain at three distinct time points. **a** At stimulus onset, the normal modes are at their largest complex amplitudes. However, due to their phases they form a destructive pattern such that the sum of their corresponding complex amplitudes is zero. **b** At the peak latency of the P1m, the largest contributions are from the two lowest-frequency modes (green and purple). These appear in nearly opposite phases, and the summed amplitude is low. **c** At the peak latency of the N1m, the normal modes with the second-lowest frequency (green) have a minute real part for all SOIs, meaning that they barely contribute to the N1m peak. Notably, the N1m peak is predominately formed by the mode with the lowest frequency (purple). The colour coding is the same as in Figs. 5 and 6, and the entire ERFs are shown in Fig. 4b

*Initial phases and mode interference*

In order to fully understand the composition of the MEG signal in terms of the normal modes, one has to appreciate that the result of a linear superposition of damped oscillations does not only depend on the amplitude of each contribution, but also on the corresponding phase. We can see from Eq. (12) that a single oscillating normal mode leads to a complex contribution of the form $r_n(t) = c_n\kappa _n\exp (\lambda _n t)$ to the ERF. For a complex conjugate pair $\lambda _n$ and $\lambda _{n+1} = \overline{\lambda _n}$, we obtain the real contribution to the MEG of the form

14 $$\begin{aligned} \begin{aligned} r_n(t) + r_{n+1}(t)&= 2 \hbox {Re}(r_n(t)) \\&= 2|c_n|\kappa _n\exp (\gamma _n t)\cos \left( \omega _nt +\arg (c_n)\right) . \end{aligned}\nonumber \\ \end{aligned}$$

Summing up such oscillatory terms with different initial phases $\arg (c_n)$ and different angular frequencies $\omega _n$ leads to complicated interference patterns, where terms at different time points may add up or cancel each other. This is illustrated in Fig. 7, which shows the amplitude and the phase of each normal mode at three time points: at stimulus onset, at the peak latency of the P1m, and at the peak latency of the N1m. Each dot represents the contribution $r_n(t)$, while the corresponding complex conjugate $r_{n+1}(t)=\overline{r_n}(t)$ is omitted. At stimulus onset, the leading modes (purple and green) have almost opposite phases such that their real parts cancel out each other (Fig. 7a). The contributions from the higher-frequency modes (blue, red) are negligible. The same holds for the P1m peak shown in Fig. 7b, where the two leading modes have very similar amplitudes but almost opposite phases. Therefore, their overall contributions are quite small, explaining why the P1m has a relatively low amplitude even though the underlying normal modes are near their extrema. After the P1m, a constructive superposition of the first and second mode starts to emerge. This superposition builds into a large-amplitude N1m (Fig. 7c). This shows that the main frequency component of an ERF can be explained as a beating frequency, that is, the frequency difference between the two leading modes. Moreover, the N1m emerges as late as it does because the leading modes are initially in opposite phase. This means that the N1m should not be interpreted as a delayed response produced by some dedicated N1 generator. This is underlined also by the fact that the activity in the core area peaks at a much earlier latency than the ERF response (see Fig. 2). The results shown in Fig. 7 highlight the fact that ERF generation is a result of a complex interplay between the spatial and temporal structure of the AC response given by the mode spectrum and the corresponding input efficiencies. Additionally, the spatial shapes of the normal modes determine their different MEG efficiencies.

**Fig. 8:**
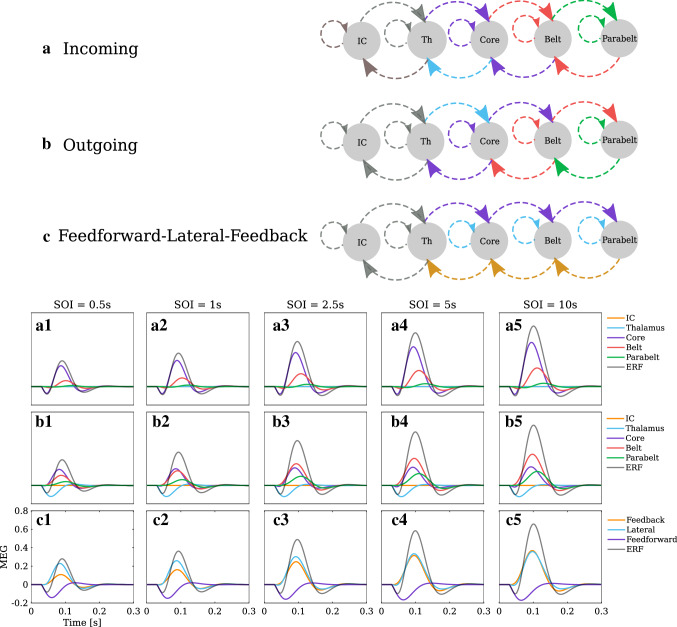
ERF contributions by connection type and area. **a1–a5** When inspecting the contributions to the ERF according to incoming connections (equivalent to source modelling), the relative contributions from each area remained the same across SOI, and each contribution was scaled similarly by the adaptation process. The main contributors to the ERF are the core (purple) and the belt (red). **b1–b5** The contributions according to outgoing connections present a different picture. Here, the belt (red) and the parabelt (green) contributions show the strongest adaptation. They both increase steeply with increasing SOI, whereas the thalamus (blue) and core (purple) contributions remain relatively stable. **c1–c5** Feedback connections (orange) contribute to the ERF with a strongly adapting component which grows as a function of SOI. In contrast, the contributions from feedforward connections (purple), responsible for the P1m, show only weak adaptation. The contribution from lateral connections (blue) displays intermediate adaptation. Therefore, the adaptation of the N1m is largely due to the adaptation of the feedback connections

### Adaptation of ERFs in terms of different types of connections

We have shown above that ERF responses can be represented as a superposition of the contributions of different normal modes. Alternatively, we can decompose the ERF according to the physiological structure in our model illustrated in Fig. 1b. Recall that according to Eq. (11), MEG signals arise as a weighted sum of contributions from individual connections in $W_{\mathrm{ee}}$ and $W_{\mathrm{ei}}$. These connections can be viewed in three ways: First, each area can be characterised in terms of the incoming connections received by that area (Fig. 8a). Second, we can describe each area according to its outgoing connections, those originating from the area (Fig. 8b). Third, connections can be categorised as feedforward (leading away from the midbrain), feedback (leading towards the midbrain), and of the lateral (intrafield) type (Fig. 8c). In terms of the connection matrices, the rows of $W_{\mathrm{ee}}$ and $W_{\mathrm{ei}}$ represent incoming connections per area and the columns represent outgoing connections per area. The diagonal elements of $W_{\mathrm{ee}}$ and $W_{\mathrm{ei}}$ are the lateral connections, and the elements below and above the diagonal of $W_{\mathrm{ee}}$ represent feedforward and feedback connections, respectively. Equation (11) enables us to break down the MEG signal into the contributions from these different types of connections.

Figure 8a1 to a5 show the decomposition of the simulated ERFs (grey) into the contributions of each area according to the incoming connections (Fig. 8a). This is equivalent to “source modelling”, looking at the contribution that the activity in each area directly contributes to the ERF in virtue of it generating a magnetic field. Note that we are assuming that the ERF is blind to activity in IC and thalamus. For all SOIs, the core area (purple) is the sole generator of the P1m and it is also the largest contributor to the N1m. The core (purple) and the belt (red) together account for almost the entire ERF, including the P2m, whereas the contribution of the parabelt (green) is minute. Note that the ratio between the magnitude of the belt and the core contribution decreases with decreasing SOI. Further, the simulations reveal an increase in the latency of the contribution to the N1m as one moves from the core to the belt and then to the parabelt.

Figure 8b1 to b5 show how the outgoing connections (Fig. 8b) contribute to the ERF (grey). These results look at the indirect contribution that each area makes via its output to other areas and to itself. Connections originating in the thalamus (blue) drive activity in the core through feedforward connections and thereby contribute to the P1m response. For the short SOIs of 0.5 s and 1 s, the core (purple) makes the largest contribution to the N1m, whereas for the longer SOIs of 2.5 s, 5 s, and 10 s, the belt’s contribution is the largest (red). The parabelt (green) with its long peak latency remains the weakest contributor to the N1m throughout all SOIs.

Figure 8c1 to c5 show how the input arriving via feedforward (purple), feedback (orange), and lateral (blue) connections contribute to the ERF. We assume that feedforward and lateral connections drive currents away from the cortical surface, whereas feedback connections drive currents towards the surface (Ahlfors and Wreh 2015; Ahlfors et al. 2015). Therefore, feedforward and lateral connections account for the P1m and P2m deflections, whereas the feedback and the inhibitory connections are responsible for the N1m. A clear pattern can be seen: the contributions driven by the feedforward and lateral connections grow only a little as SOI is increased from 0.5 s to 10 s. In contrast, the contribution from the feedback connections show strong adaptation, with a three-fold increase in magnitude over the SOI range. This differential in adaptation behaviour explains why the P1m has a weak SOI dependence and why the N1m shows the strongest adaptation.

### The role of network structure in ERF adaptation

To inspect whether the anatomical structure of AC impacts on adaptation, we derived the lifetime of adaptation in three versions of the AC model. The default version (network DEF) was the one described above, implementing the serial anatomical structure of AC (Hackett 2015). In the second version (network CP), we modified $W_{\mathrm{ee}}$ by adding a direct excitatory-to-excitatory connection between core and parabelt. In the third version (network TB), we included a direct connection between the thalamus and the belt. CP and TB represent steps towards full connectivity and are no longer serial networks. The inclusion of extra excitatory-to-excitatory connections in these networks alters the excitation-inhibition balance compared to that of the DEF network. Therefore, we also constructed normalised versions of networks CP and TB, where $W_{\mathrm{ee}}$ is modified such that it has the same norm as $W_{\mathrm{ee}}$ in the DEF network. The norm is defined as the sum of all matrix elements. The normalised structures CPN and TBN retained the excitation-inhibition balance of the original default network.

Simulations employed the stimulus-repetition paradigm described in Sect. 2.5. We used the traditional method for calculating adaptation lifetime (Lü et al. 1992; Lu et al. 1992). This was to measure the peak amplitude of the N1m for each SOI and then to fit the following exponential function to the measurements

15 $$\begin{aligned} P_{\mathrm{fit}}(\mathrm{SOI}) = A\left( 1-\hbox {exp}\left( -\frac{\mathrm{SOI}-t_{\mathrm{0}}}{\tau _{\mathrm{soi}}}\right) \right) . \end{aligned}$$

Here, $\tau _{\mathrm{soi}}$ is the time constant expressing the lifetime of adaptation; $t_0$ is the intercept with the abscissa, and *A* is the amplitude at which the exponential function saturates. All three parameters in Eq. (15) were fitting parameters. Note that in the model the saturation level *A* is equivalent to the peak amplitude of the response to the first stimulus, i.e., the initial state. For fitting we implemented an integral linear regression method to find suitable initial values (Jacquelin 2009) and, then, used a nonlinear regression function (nlinfit) from MATLAB (The MathWorks, version R2018b) to estimate the fitting parameters. We performed the fitting procedure not only to the N1m peak amplitudes, but also to the peak amplitudes of the state variables *u*(*t*) of the core, belt, and parabelt.

**Fig. 9:**
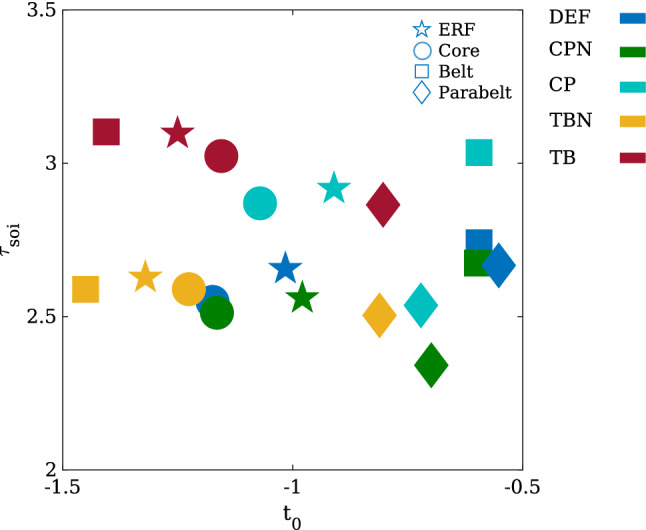
The impact of network structure and excitation-inhibition balance on adaptation. The recovery time constant $\tau _{\mathrm{soi}}$ and the intercept $t_0$ were obtained by fitting Eq. (15) to the peak amplitude of the simulated N1m (star), and of the state variables *u*(*t*) of core (circle), belt (square), and parabelt (diamond). Blue symbols show $\tau _{\mathrm{soi}}$ and $t_0$ for the default AC network (DEF, see Fig. 1). Adding a thalamocortical connection between thalamus and belt area (TB, red) has the strongest effect on $\tau _{\mathrm{soi}}$ and $t_0$. The same network with normalised balanced excitation-inhibition (TBN, yellow) leads to smaller values of $\tau _{\mathrm{soi}}$ and to little change of $t_0$. The addition of a corticocortical connection between core and parabelt (CP, cyan) leads to an increase of $\tau _{\mathrm{soi}}$ everywhere except in parabelt. However, the same network with normalised excitation-inhibition balance (CPN, green) shows smaller values of $\tau _{\mathrm{soi}}$

Figure 9 shows the fitting parameters $\tau _{\mathrm{soi}}$ and $t_0$ characterising the SOI-dependence of the peak amplitude in the case of the ERF (star) and of the core (circle), belt (square), and parabelt (diamond) state variable *u*(*t*). The results demonstrate that these parameters are sensitive to the network structure as well as to the excitation-inhibition balance. Two important observations can be made. First, the impact of the structural changes can be identified by comparisons between the default network (DEF, blue) and the normalised TBN (yellow) and CPN (green) network, which differ from DEF solely in terms of structure. Whereas $\tau _{\mathrm{soi}}$ appears to be weakly affected, with values between 2.3 s and 2.7 s, the variation of $t_0$ is stronger, covering the range from − 1.5 s to − 0.5 s. Second, the effect of the excitation-inhibition balance on $\tau _{\mathrm{soi}}$ and $t_0$ is revealed by comparisons between TBN (yellow) and TB (red) and between CPN (green) and CP (cyan). In each case, the normalised network versions TBN and CPN show less excitation than the non-normalised versions TB and CP. We see that the effect of adding excitation is to push $\tau _{\mathrm{soi}}$ up by 500 ms. In contrast, $t_0$ of core and parabelt is only weakly affected by added excitation. Taken together, Fig. 9 shows that the modification of the excitation-inhibition balance has a larger effect on $\tau _{\mathrm{soi}}$ and $t_0$ than a change of the network structure.

**Fig. 10:**
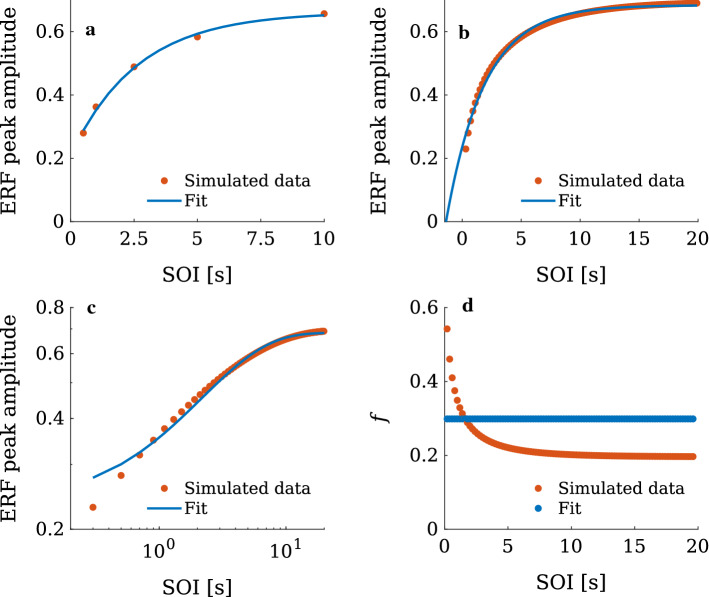
A single exponential function does not capture the recovery from adaptation. **a** The fitting function of Eq. (15) appears to offer a reasonable approximation (blue line) of the SOI-dependence of the N1m peak amplitudes (red points) when there are only a few measurement points. **b** The situation, however, is different when a much larger number of SOIs can be used for revealing the true SOI-dependence of the N1m. There is a systematic deviation between the fitting function and the peak amplitudes. **c** The *log*(*x*) transformation of the data points emphasises that there are clear differences between the data points and the fit for small and larger SOIs. **d** This deviation between the data points and the fit can be quantified by computing the local saturation rate *f*, given in Eq. (16), for any two consecutive data points. For the exponential fit, the local saturation rate *f* is constant, whereas for the simulated data points it monotonically decreases with increasing SOI and converges towards a constant value ($\approx$ 0.2), different from the *f* obtained from the fit ($\approx$ 0.3), only at large SOIs ($\ge$10 s)

### Is a single exponential function sufficient to explain recovery from adaptation?

Adaptation is traditionally quantified through fitting the single-exponential function of Eq. (15) to the peak amplitudes of the N1m. But does a single exponential actually describe the dependence of the ERF amplitude on SOI? Figure 10a indicates that this appears to be the case, at least when the number of data points is low. However, with our model we can easily generate ERF peak amplitudes for an arbitrary number of SOIs which would not be feasible experimentally. This is illustrated in Fig. 10b where 99 simulated ERF peak amplitudes (red points) are plotted as a function of SOI together with the corresponding fit (blue line). Figure 10b shows a systematic deviation of the fitting function (Eq. (15)) from these data. This deviation is highlighted in Fig. 10c where the data points in Fig. 10b are transformed by the *log*(*x*) function. Figure 10c shows that at small SOIs ($\le$1.5 s), the simulated data deviate much stronger from the fit than data at larger SOIs. This deviation might seem negligibly small. However, it indicates two major shortcomings of such exponential functions for explaining recovery from adaptation. First, the strong deviation at small SOIs, highlighted in the logarithmically scaled plot in Fig. 10c, puts a question mark on the general applicability of a single-exponential function for the description of the recovery process. Second, it questions the explanatory power of the fitting parameter $t_0$. This deviation between fit and data can be quantified more precisely by the local exponential saturation rate

16 $$\begin{aligned} f_j = \frac{F_j-F_{j+1}}{(F_{j}-F_{\mathrm{inf}})(\mathrm{SOI}_{j+1}-\mathrm{SOI}_j)} , \end{aligned}$$

where the *F*s are simulated ERF peak amplitudes and *j* is the index of the data points (red points in Fig. 10b–d). $F_{\mathrm{inf}}$ is the amplitude at which the data points saturate. It is equivalent to the ERF peak amplitude at the initial state, this being the maximum possible value the simulated ERFs can have. The results of the computation of the local exponential saturation rate for the simulated data as well as the fit are shown in Fig. 10d, which for the fitting function given in Eq. (15) always provides the constant value $1/\tau _{\mathrm{soi}}$. However, it also shows that simulated data points (red) deviate substantially from a constant value for SOIs smaller than 5 s.

In summary, the above results show that our description of the adaptation process as a collective reorganisation of the AC as a dynamical network also captures the behaviour of the N1m peak amplitude variation as a function of SOI. It turns out that for smaller SOIs this adaptation behaviour differs substantially from the widely-used single exponential function. As a consequence, if one estimates adaptation lifetimes through fitting such a function to amplitude data, the result might strongly depend on the choice of the sampling point.

## Discussion

### Main findings

We used mathematical modelling to investigate context sensitivity of auditory cortex specifically, how STSD modifies the systems dynamics of auditory cortex and how this modification becomes visible as the adaptation of the ERF associated with stimulus repetition. We took an approach similar to the one in our previous study (Hajizadeh et al. 2019) whereby the auditory cortex was characterised as a set of spatially distributed, mutually independent oscillators—normal modes—exposed through explicitly solving the state equations. Each normal mode is a global feature because it contributes to the activity of all cortical columns and depends on the anatomical structure of the entire auditory cortex. Compared to the traditional view that the ERF arises out of spatially discrete, local generators, our approach offers an alternative: the ERF is generated by a set of processes where each one is distributed over the whole network of the auditory cortex. That is, we go beyond describing activity of auditory cortex in terms of amplitude variations of the activity of spatially fixed cortical columns. In contrast, in our account, the activity of auditory cortex is described as oscillations of normal modes which are spread over the whole AC anatomy. In this view, the activity of an individual column emerges out of the interplay between the amplitudes, frequencies, and phases of all the normal modes of the system. An analogy for understanding stimulus-elicited brain activity in terms of normal modes is to think of a pebble thrown into a still pond. The ripples formed on the water’s surface are functions of the size, shape, and the speed of the pebble (i.e., the stimulation), but also depend on the size, shape, and water quality of the pond (i.e., the system). If new pebbles are thrown before the previously formed ripples fade away, different locations on the pond surface oscillate based on the interference pattern of the old and the new ripples. The dynamics of AC as a spatially extended structure is no different than the pond. By stimulating AC, the activity propagates in time and space through the system. This propagation is then a function of the stimulus and of the anatomical structure of AC. The activity at each AC location oscillates as a result of the interference pattern of the normal modes which are spread over the whole AC (see Fig. 5k–o). That is, each location participates in multiple, overlapping oscillations that are occurring both in time and space. The MEG device then projects these oscillations onto single magnetic field values.

Expanding from Hajizadeh et al. (2019), the current study addressed adaptation due to stimulus repetition through the introduction of dynamical synapses to the model. This was achieved through two manoeuvres: First, we derived complete solutions to the system of Eqs. (1) and (2) even in cases where the connections are asymmetric, such as when STSD is in operation. Second, we used time-scale separation of the dynamics of STSD (Eq. (3)), exploiting the relative slowness of recovery from STSD in comparison to the fast evolution of the state variables (Eqs. (1) and (2)). As a result, we were able to describe the adapting auditory cortex as a set of normal modes modulated by the stimulation. In this approach, all possible system trajectories are solved in one go rather than simulated one at a time.

The current model replicates the experimentally observed adaptation of the ERF resulting from stimulus repetition. This can be observed as, for example, the peak amplitude of the N1m increasing monotonically as a function of SOI, roughly according to an exponentially saturating function. The N1m can be understood as an interference pattern of the superimposed normal modes, and in this view, its adaptation is explained as resulting from the modulations not only of the amplitudes but also of the angular frequencies $\omega _n$ of the normal modes. Indeed, adaptation should be seen as a complete reorganisation of the AC network where the reduction of the N1m amplitude is a by-product of the stimulation shifting the dispersion of the angular frequencies $\omega _n$ and decay rates $\gamma _n$ of the normal modes.

While the adaptation of the N1m can be accounted for by changes in the underlying normal modes, the N1m is only a single landmark in the ERF. To gain a more complete view of how the dynamics of the system are modulated by previous stimulation, we inspected how the normal modes change as a function of SOI in the adapted state. The main characteristics of a normal mode are its frequency $\nu$ and decay rate $\gamma$, as displayed in Fig. 5f–j. Two observations could be made. First, there is a neat dispersion of the normal modes in the $\nu$-$\gamma$ plane where they land on a monotonically increasing curve, so that the higher the frequency, the faster the decay rate. Second, the effect of STSD is to push the normal modes upwards on this dispersion curve with respect to the initial state, this effect being larger the smaller the SOI is. We can thus view adaptation of the ERF in a novel way: each incoming stimulus leaves a memory trace behind as a change in the system dynamics so that all normal modes are shifted towards higher frequencies and faster decay rates. During the interval between successive stimuli, the memory trace decays so that the normal modes slide back towards their respective unadapted states. The degree to which a normal mode contributes to the MEG signal depends on two efficiency factors (see Eq. (13) and Fig. 6) and their product determines the actual contribution made by the normal mode to the MEG signal. Adaptation due to stimulus repetition shows up as a modulation of the efficiencies, which can either expand, shrink, or remain the same as SOI is modulated, depending on the normal mode.

Further, we found that adaptation is a network effect that depends not only on STSD. Namely, changing the anatomical structure of the network and/or the balance between excitation and inhibition led to marked changes in the lifetime of N1m adaptation, even though the parameters of STSD were kept constant. Also, inspecting the individual contributions to the ERF from the various areas, we found that the lifetime of adaptation varied across anatomical location.

### Adaptation of the N1m: what are we measuring?

The current model of the AC replicates well the experimentally observed SOI-dependence of the ERF (see Fig. 4), and all main features of the waveforms can be traced back to the distinct contributions of the individual cortical areas, as laid out in Fig. 8. The identical rising slopes of the waveforms derive from the core, which provides the largest contribution to the MEG waveform at all SOIs and is also the main source of variation of the magnitude of the N1m peak. The belt also contributes to the overall amplitude, especially at longer SOIs. The magnitude of the parabelt contribution to the ERF is negligible at all SOIs. However, this disguises the influence of the parabelt on the overall dynamics of the system, as shown by our previous results (Hajizadeh et al. 2019): small changes in the connection values within the parabelt result in sizeable modulations of the N1m response, even though the parabelt’s direct contribution to the N1m is minimal. Further, we found that the adaptation of the N1m is largely due the adaptation of the contribution generated by the feedback connections, while the feedforward and lateral (intrafield) components remained relatively stable across SOI (Fig. 8).

We emphasise that in the model, the ERF signal is the weighted superposition of multiple normal modes as distributed over the core, belt, and parabelt (Fig. 5). Therefore, the N1m peak represents an event in an interference pattern rather than anything real. That is, the peak of the N1m at about 100 ms is incidental in the sense that it does not represent the peak of any normal mode nor that of the activity of any individual area in the AC. In consequence, observing adaptation as SOI-related changes in the N1m on its own reveals very little of how the underlying dynamics are changing. To understand what is driving N1m adaptation, we investigated how the interference pattern of the normal modes changes as SOI is varied.

Figure 5 shows the ERF in terms of its normal mode components, which paint a consistent pattern across the five SOI conditions. These components come in a variety of peak amplitudes and latencies, and they can also have opposite phases, which contributes to mutual cancellations when the modes are summed up to the ERF signal (see also Fig. 7). Interestingly, each normal mode reaches its global absolute peak well before the N1m even starts to emerge. Yet, in the ERF, the N1m is the dominating wave. This is due to the low-frequency modes largely cancelling each other out during the first 70 ms, when the modes reach their global extrema. Because of this cancellation effect, the P1m response has a low amplitude, even though it occurs when the normal modes are at their most vigorous (see Fig. 7). Subsequently, when all the normal modes are well into their decay phase, the N1m emerges as an interference pattern of the two low-frequency normal modes with the highest amplitudes but opposite phases.

On the above basis, we can understand the adaptation of the N1m in terms of the behaviour of the two dominating normal modes of auditory cortex. That is, adaptation arises out of two factors which contribute unequally in different SOI ranges. As shown in the left column of Fig. 5, with the fast stimulation rates (SOIs of 0.5 s, and 1 s), the normal modes are clearly attenuated in amplitude compared to their counterparts in the 10-s SOI condition (representing the unadapted state). Furthermore, mirroring the adaptation of the N1m (Fig. 4), this attenuation is much larger when SOI is 0.5 s than when SOI is 1 s. In contrast, when the SOI is larger (2.5 s, 5 s, and 10 s), the amplitudes of the normal modes become insensitive to changes in SOI. Indeed, the normal mode with the lowest frequency (purple curve in Fig. 5) *decreases* slightly in amplitude as SOI is increased from 2.5 s to 10 s, while the N1m grows in amplitude. In this case, the attenuation of the N1m is explained by the changes in the frequencies rather than the amplitudes of the two dominating normal modes: the mode with the largest absolute magnitude (green curve in Fig. 5) remains relatively stable in terms of amplitude and frequency, while the mode with the second-largest magnitude but opposing phase (purple curve in Fig. 5) increases in frequency as SOI decreases. Because of this difference in their frequency behaviour and phase, the dominant normal modes sum up to an N1m response that decreases with decreasing SOI.

The adaptation of the N1m is usually described with the single exponentially saturating function of Eq. (15). This captures the behaviour of the peak amplitude of the N1m, namely, its initially rapid increase as a function of SOI followed by a levelling off at longer SOI. This description might be adequate when the number of data points is low (Fig. 10a). However, our model predicts that the true dependence of the N1m peak amplitude on SOI is insufficiently described by a single exponential function (Fig. 10b–d). This could be a consequence of the N1m peak amplitude being determined by two different normal-mode properties, viz. due to normal mode modulations at short SOIs and to frequency modulations at longer SOIs. Thus, adaptation lifetime as estimated with the single exponential might work better as a relative rather than an absolute measure. Even if it fails to describe amplitude behaviour at short SOIs, it still allows one to compare lifetimes across experimental conditions, brain regions, and subjects.

### Linking ERF adaptation to the network structure of auditory cortex

Physiological studies usually consider the link between STSD and adaptation from the point of view of single-unit dynamics (Ulanovsky et al. 2004; Wehr and Zador 2003, 2005). When one observes a global, spatially-averaged measure of neuronal activity such as the ERF, our results indicate that STSD is not the only factor shaping adaptation. Instead, adaptation becomes a system property, modulated by anatomical structure. This is unsurprising given that all normal-mode properties (angular frequency $\omega$, decay rate $\gamma$, phase, spatial structure) arise from the coefficient matrix in Eq. (4), which in turn depends on the anatomical structure of AC as expressed in the weight matrices. We varied the structure of the original AC model (Fig. 1) by adding shortcut connections from the thalamus to the belt, or from the core to the parabelt. We also varied the balance between excitation and inhibition. All other aspects of the model were kept untouched, including the time constants of the state equations and those of STSD. Nevertheless, the structural modulations in themselves caused sizeable variations in the way the N1m became adapted by repeated stimulation: the parameter $t_0$ varied between -1.4 and -0.9 s, and $\tau _{\mathrm{soi}}$, the adaptation lifetime, varied between 2.5 and 3.1 s. In general, changing the excitation-inhibition balance by adding excitatory connections increased the lifetime of adaptation.

We also derived the individual contributions from each cortical area (core, belt, parabelt) to the overall ERF in order to inspect whether adaptation has a spatial variation. This was done for all the structural modulations studied above. Experimentally, this would be equivalent to applying source modelling to the ERF signal to tease out the contributions from various areas of cortex. We found that, in general, the adaptation of the ERF did not coincide with that of any of the contributions of the individual areas in terms of $t_0$ and $\tau _{\mathrm{soi}}$. The lifetime of adaptation tended to be some 200 ms shorter in the core than in the belt. The parabelt had the shortest $\tau _{\mathrm{soi}}$, except in the default AC model, where the belt and the parabelt had similar lifetimes of adaptation. These results agree qualitatively with those of Lu et al. (1992), who found that the contribution of primary auditory cortex to the N1m has a shorter lifetime of adaptation (by seconds) than the contribution from association areas. A similar pattern was observed by Uusitalo et al. (2006) in visual cortex, where the adaptation lifetime increases (by seconds) as one moves further away from primary visual cortex. The spatial variation of adaptation lifetime produced by our model is much smaller than that found experimentally, and the factors determining the size of this effect will be addressed elsewhere.

Importantly, the above spatial variation of adaptation belies a much stronger effect of anatomy on adaptation. This is evident in Fig. 8b1–b5 which break down the ERF according to the connections originating from each area. For each area, this measure is proportional to the output emanating from that area, that is, to the firing rate multiplied by the connection strength. It can therefore be interpreted as the de facto impact that the area has on its neighbours and on itself. The impact of the core remains stable, increasing only around 10 % over the SOI range. In stark contrast, the impact of the belt and parabelt is highly sensitive to stimulation rate: it increases linearly by a factor of three as one increases SOI from 0.5 s to 10 s. That is, there is hardly any adaptation present in the impact that the core has, while the impact of the belt and the parabelt exhibits strong adaptation. Although we did not determine $\tau _{\mathrm{soi}}$ for these impacts, it is evident that on this metric, adaptation lifetime is orders of magnitude larger in the belt and parabelt than in the core. We emphasise that these effects are not directly visible in the ERF, even if one measures $\tau _{\mathrm{soi}}$ separately for each area (corresponding to source modelling). Further, the presence of a spatial gradient of adaptation lifetime would have interesting implications for understanding memory in auditory cortex. Namely, the anatomy of AC may serve as a temporal map, where secondary areas, in functional terms, hold information over several seconds and where the core integrates the current signal with the memory-laden feedback from the secondary areas. This style of temporal mapping might be crucial for the processing of auditory signals with a complex spectrotemporal structure and warrants further investigation.

### Comparison to other models of auditory cortex and ERF generation

The role of STSD in AC dynamics has been investigated in a number of previous modelling studies. Loebel et al. (2007) developed a model of the primary AC where each iso-frequency column was described as a network with Wilson and Cowan (1972) dynamics and with STSD. The model can account for multiple experimental findings such as the frequency tuning curves of neurons and the dependence of forward masking in two-tone stimulation on the temporal separation between the tones. In a later work, Yarden and Nelken (2017) demonstrated that this model is able to replicate stimulus-specific adaptation. Goudar and Buonomano (2014) modelled primary auditory cortex with simulated spiking neurons and found that short-term synaptic plasticity accounted for context-dependent suppression and enhancement of the response to the second tone in a two-tone paradigm. Similar order-selectivity in responses to vocalisation stimuli was found by Lee and Buonomano (2012), who modelled a single cortical column with spiking neurons. Again, STSD accounted for the neurons responding differentially to vocalisations presented in the forward and reverse directions. Wang and Knösche (2013) replicated the adaptation of the N1m due to stimulus repetition in a model of a single cortical area, where each unit was an expanded version of the neural mass model of Jansen and Rit (1995), which describes interactions between neurons in the granular, supragranular, and infragranular layers. The model included STSD of excitatory synapses, and good agreement with experimental data was achieved by adjusting the inter- and intralaminar connections via Bayesian inference.

The above studies are thematically related to the current approach in that synaptic depression is shown to account for experimental data. However, they are limited to describing either primary auditory cortex or a single column with a relatively high resolution and, with the exception of Wang and Knösche (2013), replicate single-unit activity. In contrast, our approach for understanding ERF generation is to capture the dynamics of the whole of AC, rather than that of a single field. To this end, we implemented the serial core-belt-parabelt structure in a model that anchors ERF generation to spatially distributed normal modes. Therefore, our approach diverges from the above studies in terms of how AC is described and in the kind of explanation given for the data. We note that while we employed the extreme low resolution of describing each area as a single unit, our approach is not wedded to any particular spatial resolution. Our previous studies (Hajizadeh et al. 2019, 2021) used spectral methods similar to the ones employed here (though without STSD) for investigating the normal modes in a system of 240 units representing cortical columns distributed over subcortical areas and 13 tonotopically organised cortical fields. Importantly, the current results open up the possibility of applying spectral methods for studying STSD modulation of AC dynamics in an expanded model with a much higher spatial resolution than here. We note that boosting the number of units adds very little computational cost. Numerical simulations based on Eqs. (1), (2), and (3) are computationally expensive for large coupled networks and can be error-prone due to numerical errors and sensitivity to initial conditions. The spectral approach championed here, in comparison, is computationally fast and readily unveils the dependencies between the systems parameters and the solutions.

Probably the most influential style of neural mass modelling is Dynamic Causal Modelling (DCM) introduced by Friston et al. (2003). This estimates the coupling between different brain regions and how this is modulated by stimulation. Each region is described by a biophysical neural mass model, and Bayesian inference is then used for the parameter estimation to identify the best model to explain the experimental data. The DCM approach has been applied to ERPs and ERFs (see, for example, David et al. 2006; Garrido et al. 2007; Kiebel et al. 2006, 2009), the hemodynamic response of functional magnetic resonance imaging (fMRI) (for example Friston et al. 2019; Stephan et al. 2007) and the neurovascular coupling underlying combined fMRI and MEG/EEG data (for example, Friston et al. 2019; Jafarian et al. 2020). When applying the method to ERPs and ERFs, the biophysical model is based on the Jansen and Rit (1995) approach. Estimates are then derived for intrinsic connections within brain regions as well as for feedforward, feedback, and lateral connections between brain regions.

While our spectral approach and DCM both seek to explain evoked responses, the two diverge on a number of points. First, DCM aims to explain single-subject ERF data in terms of connection strengths between discrete sources. The method is essentially a refinement of source localisation, where source location is complemented by information about the coupling strength between the sources. In contrast, our approach regards individual sources and their connections as only part of the explanation. From the point of view of the system dynamics, the spatially distributed normal modes provide a more revealing account of the ERF. Of course, whether normal modes can be observed experimentally is an interesting question beyond the scope of the current study. Second, while the spatial resolution in the current model was at one unit per cortical area—the same as in Garrido et al. (2009)—spatial resolution is not a limiting issue in our approach, as discussed above. In comparison, DCM places bounds on the size of the network that can be used for modelling brain activity. Namely, increasing the number of units leads to an exponential increase in the number of connections, which are all free parameters to be estimated. As pointed out by Garrido et al. (2009), this results in inter-subject variability becoming larger, making it hard to establish patterns of coupling changes across subjects. Third, DCM is designed to be used with discrete source models of the ERP and ERF, with each area represented by a single unit of the biophysical model. In contrast, the number of units per area is not limited in our approach, and is determined by the phenomenon to be explained. For example, modelling frequency interactions (e.g., the frequency mismatch response) with one unit per area can be done with DCM (Garrido et al. 2007) but with our approach, an expansion of the model to include tonotopic maps in each area would be required. This essentially reflects the fundamentally mechanistic nature of our modelling style.

## Outlook

There is scope for expanding the current model in several ways. First, as mentioned above, the modelling of cross-frequency effects would require the implementation of tonotopic maps, such as in the previous versions of the model (Hajizadeh et al. 2019, 2021; May and Tiitinen 2013; May et al. 2015). This would allow one to gain fresh insight into the generation of the MMN and SSA in terms of normal modes. Time constants for SSA have been reported to occur on multiple time scales (Ulanovsky et al. 2004), and there might be scope to study this with our modelling approach, in view of our finding that anatomical structure in itself introduces variations to adaptation lifetime, even when the time constants for STSD are spatially homogeneous.

Second, while the core-belt-parabelt structure of AC is a common feature among the auditory cortices of mammals (Hackett 2015), there is a wide variety in the size and organisation of AC areas across species, and the functional consequences of this variety are unknown. Hence, a logical next step would be to expand the model towards more realistic structures of auditory cortex of different species, and to investigate to what extent adaptation is a network effect whose cross-species variations can be explained in terms of differences in the anatomical structure of the AC. Further, one might be able to use the current methodology as a tool for exploring the currently unknown organisation of the human auditory cortex. One possibility might be to combine the current methods with DCM by using the current model as the biophysical DCM model. The free parameters would be the STSD time constants as well as the parcellation of the core, belt, and parabelt into individual fields, each one represented by a unit of the model. While the STSD would presumably be subject-specific, the parcellation would be fixed across subjects.

Lastly, while the brain is usually regarded as highly non-linear, it might turn out to be a clandestine self-lineariser, using STSD as a mechanism which pushes the system dynamics into the linear range. This might allow the transition from chaotic regions into states where normal modes appear. As Kerschen et al. (2009) pointed out, normal modes of linear systems differ from those of non-linear systems in that they are decoupled from one another. This means that they have two special properties: (1) invariance, whereby several normal modes can coexist in the system at the same time without modulating each other; (2) modal superposition whereby the oscillations of a unit is a linear combination of individual normal modes. We suspect that these properties could have functional benefits: normal modes of linear systems, each with its own spatial profile, could function as stable and overlapping representational tokens supporting population coding, where each neuron can take part in representing more than one thing at the same time. This style of representation might aid processes such as sensory binding and attention control. Namely, the features of sensory stimuli are represented in a distributed fashion, in specialised regions across cortex, yet this information is melded together into unitary percepts. Sensory binding refers to this process of melding, and it seems to involve the long-distance synchronisation of the spatially disparate neuronal populations representing the individual features (Bertrand and Tallon-Baudry 2000; Ghiani et al. 2021). Selective attention is likewise associated with coherence: the cortical neurons representing the attended stimulus produce enhanced, synchronised gamma-band oscillations (Fries 2015). Normal modes could provide instantaneous coupling needed in binding and attention, allowing for individual cortical neurons separated by long distances to become synchronised even without direct connections between them. The dominant normal mode in cortex might then correspond to the attended, perceptually bound stimulus. A corollary of this is that functional cell assemblies generated in this fashion cannot be predicted just by observing the anatomical connections between the cells. This widens the view onto the generation of cell assemblies, which are usually understood in terms of communication between senders and receivers (Hahn et al. 2019) and strong interconnections arising out of Hebbian learning (Gerstein et al. 1989; Wennekers et al. 2003). With normal modes, the synchronisation between two cells depends more on the afferent stimulation and system dynamics than it does on the strength or quality of the interconnection. Moreover, normal modes might provide the mechanism whereby weakly connected populations can synchronise, before Hebbian learning has had time to take effect.

## Data Availability

The data analysed during the current study is available from the corresponding author on reasonable request.

## Code availability

The code used in the current study is available from the corresponding author on reasonable request.
